# Theoretical background, first stage development and adaptation of a novel Integrative Adapt Therapy (IAT) for refugees

**DOI:** 10.1017/S2045796019000416

**Published:** 2019-08-23

**Authors:** A. K. Tay, M. A. A. Miah, S. Khan, M. Badrudduza, K. Morgan, S. Balasundaram, D. Silove

**Affiliations:** 1 School of Psychiatry, University of New South Wales Sydney, Australia; 2 Perdana University-Royal College of Surgeons in Ireland (PU-RCSI) School of Medicine, Selangor, Malaysia; 3 Department of Psychology, Jagannath University, Dhaka, Bangladesh; 4 Health Unit, United Nations High Commissioner for Refugees (UNHCR), Kuala Lumpur, Malaysia

**Keywords:** Cognitive therapy, common mental disorders, other psychosocial techniques/treatments, post-traumatic stress disorder, trauma, refugees, post-conflict, psychotherapy

## Abstract

**Aims:**

Refugees are confronted with the task of adapting to the long-term erosion of psychosocial systems and institutions that in stable societies support psychological well-being and mental health. We provide an overview of the theoretical principles and practical steps taken to develop a novel psychotherapeutic approach, Integrative Adapt Therapy (IAT), which aims to assist refugees to adapt to these changes. This paper offers the background informing ongoing trials of IAT amongst refugees from Myanmar.

**Methods:**

A systematic process was followed in formulating the therapy and devising a treatment manual consistent with the principles of the Adaptation and Development After Persecution and Trauma (ADAPT) model. The process of development and refinement was based on qualitative research amongst 70 refugees (ten from West Papua and 60 Rohingya from Myanmar). The therapeutic process was then piloted by trained interventionists amongst a purposively selected sample of 20 Rohingya refugees in Malaysia.

**Results:**

The final formulation of IAT represented an integration of the principles of the ADAPT model and evidence-based techniques of modern therapies in the field, including a transdiagnostic approach and the selective use of cognitive behavioural treatment elements such as problem-solving and emotional regulation techniques. The steps outlined in refining the manual are outlined in relation to work amongst West Papuan refugees, and the process of cultural and contextual modifications described during early piloting with Rohingya refugees in Malaysia.

**Conclusions:**

IAT integrates universal principles of the ADAPT model with the particularities of the culture, history of conflict and living context of each refugee community; this synthesis of knowledge forms the basis for participants gaining insights into their personal patterns of psychosocial adaptation to the refugee experience. Participants then apply evidence-based techniques to improve their capacity to adapt to the serial psychosocial changes they have encountered in their lives as refugees. The overarching goal of IAT is to provide refugees with a coherent framework that assists in making sense of their experiences and their emotional and interpersonal reactions to the challenges they confront within the family and community context. As such, the principles of a general model (ADAPT) are used as a springboard for making concrete, manageable and meaningful life changes at the individual level, a potentially novel approach for psychosocial interventions in the field.

## Introduction

Over 68.5 million persons worldwide have experienced forced displacement in contemporary times, the largest number since World War II (United Nations High Commissioner for Refugees, 2018). The impact of the refugee experience on mental health can be direct, that is, via exposure to traumatic events and daily stressors, or indirect, as a consequence of the longer-term erosion of the psychosocial systems and institutions which in stable societies support psychological well-being and mental health (Tay and Silove, 2016). Disruptions to these background psychosocial systems, and the capacity to repair them, vary according to the context and phase of the refugee experience, extending from mass conflict and persecution to displacement, transition and resettlement. As anticipated, traumatic experiences, ongoing stressors and the disruption of background systems of psychosocial support interact in complex ways to shape mental health responses amongst refugees (Tay *et al*., 2015*a*).

To date, brief psychotherapeutic interventions applied to refugees have focused on past trauma and ongoing stressors, drawing largely on mainstream principles derived from cognitive behavioural therapies (CBT) and associated techniques. We describe the early development of a psychotherapeutic approach which focuses on the stressors and adaptive difficulties caused by the undermining of the core psychosocial systems during the course of the refugee experience. Our approach, referred to as Integrative Adapt Therapy (IAT) is based on the Adaptation and Development After Persecution and Trauma (ADAPT) model (Silove, 2013). In this article, we describe the theoretical underpinnings of IAT, the formulation, development, refinement and cultural adaptation of a treatment manual to guide the intervention, and the steps we have pursued in piloting the therapy. This long-term background work has led to the initiation of clinical trials using IAT amongst refugees from Myanmar in Malaysia and Bangladesh. These trails are close to completion and the results will be reported in subsequent articles.

### Theoretical underpinnings of IAT

Refugees and conflict-affected populations manifest a wide range of both normative and maladaptive emotional, behavioural and social reactions to the combination of challenges they face including exposure to trauma, ongoing living difficulties and loss of the psychosocial support systems that usually assist in maintaining the psychological equilibrium in the individual (Silove *et al*., 2017*a*). It is now recognised that the psychiatric outcomes of adversity experienced by refugees can be diverse, often exhibiting a pattern of comorbidity in which posttraumatic stress disorder (PTSD), the diagnosis that has attracted most attention in the refugee field, is one of several overlapping symptom constellations. (Steel *et al*., 2009; Turrini *et al*., 2019). A transdiagnostic approach therefore is most appropriate in this field, ideally including both conventional diagnostic categories as well as relevant indigenous or culturally-specific syndromes reflecting local idioms of distress (Hinton and Lewis-Fernandez, 2010; Silove *et al*., 2017*a*).

The ADAPT model organises the psychosocial disruptions caused by mass conflict and displacement into five core pillars supporting safety/security; interpersonal bonds and networks; justice; identities and roles; and existential meaning (Silove, 2013). Undermining or disruption of each pillar is associated with core psychosocial reaction patterns both at the individual and collective level. Chronic or recurrent threat to safety and security (Pillar 1) generates fear and anxiety, and when the threat extends beyond the individual's capacity to adapt, manifests in overt symptoms of PTSD and other forms of clinical anxiety (Tay *et al*., 2015*d*); multiple traumatic losses and separations (Pillar II) lead to grief and when unmitigated, result in complicated forms of bereavement and extreme separation anxiety which in turn affect ongoing relationships (Silove *et al*., 2010; Tay *et al*., 2016*a*); exposure to gross human rights violations (torture, sexual abuse, massacres) often provokes an intense sense of injustice (Pillar III) associated with normative feelings of anger, a response that can become dysfunctional when anger expresses itself in inappropriate aggression (Rees and Silove, 2011; Tay *et al*., 2015*c*; Silove *et al*., 2017*b*); loss of roles and identities (Pillar IV) can challenge the sense of identity and at the extreme, feelings of marginalisation and anomie (Tay *et al*., 2019); and disruption of systems of meaning in the social, cultural, political, spiritual and religious domains (Pillar V) challenges the individual to re-evaluate established belief systems which in turn can lead to a sense of incoherence and existential despair (Basoglu *et al*., 2005).

Mass conflict commonly erodes the ADAPT pillars simultaneously leading to complex and overlapping psychosocial and psychiatric responses (Tay *et al*., 2015*a*) which are shaped by the culture and context. Moreover, the process of adaptation is dynamic, each stage of the refugee process creating new challenges to the ADAPT system and requiring flexibility in the way individuals and their communities respond in the context. For each person, in therapy it is important for the general framework of the ADAPT model to be applied in a way that particularizes and makes concrete the experience and psychological reactions of the individual. In that way, the model confers a specific set of meanings to the lived experience of the person while at the same time making reference to common experiences with others in the community who have faced similar challenges. IAT therefore aims to provide a framework of meaning to the life experiences of refugees so that their emotional and behavioural reactions make sense to them based on the general principles provided by the ADAPT model. IAT therefore attempts to reconcile idiographic and nomothetic perspectives by grounding the individual, subjective experience of the person within a broader framework of understanding that is relevant to refugees in general and in which conventional categories of mental disorder retain a meaningful place (Tay and Silove, 2016). Further, because a fundamental tenet guiding IAT is that the intervention requires adaptation to each context, it is encouraged that the approach be modified to ensure its relevance to each context and culture. Hence its use requires a process of preliminary qualitative work in each setting in which the nature and extent of erosion of the ADAPT systems must be evaluated. For example, a qualitative study of adolescent refugees securely resettled in Australia found that pillar IV (roles and identities) of the ADAPT model was most relevant, consistent with the age of development and context in which these young people lived (McGregor *et al*., 2016). In extreme conditions of ethnic cleansing, deprivation and marginalisation, such as amongst Rohingya refugees, all five ADAPT pillars were found to be severely disrupted.

### Correspondence between the ADAPT pillars and common mental disorders in refugees

Research has provided support for the importance of all five constituent pillars of the ADAPT model; the impact of life-threatening events (Pillar I) (Tay *et al*., 2016*b*), traumatic loss (Pillar II) (Tay *et al*., 2015*d*, 2016*a*) and the sense of injustice (Pillar II) (Tay *et al*., 2017) have each been linked to common mental disorders (CMDs), specifically PTSD, complicated grief and explosive anger, respectively (Tay *et al*., 2016*c*, 2016*d*). Nevertheless, although there is a degree of specificity in these relationships, patterns of overlap are common, given that the ADAPT pillars often are undermined simultaneously and there is a high level of comorbidity involving CMDs and related psychological reactions amongst refugees.

### Distinctions between IAT and cognitive behavioural treatments

As outlined, IAT is distinctive in focusing explicitly on the five psychosocial pillars of the ADAPT model, allowing refugees to make meaningful connections between past experiences of disruptions (including traumatic events), their personal responses (emotional, cognitive, behavioural, interpersonal), their perceptions of and capacity to cope with the current situation, and their anticipation of coping with future challenges. The treatment goal is to draw on the enhanced understanding of these issues to develop adaptive coping skills in order to address present problems and help navigate future challenges. Although often extending beyond the core objective, extant approaches to CBT, including trauma-focused CBT, tend to focus primarily on addressing maladaptive cognitive and behavioural patterns of responding to trauma and its aftermath. The techniques used to do so are based on general principles of CBT and may not be fully contextualised in relation to the lived experience, cultural background and experience of mass violence that characterise the refugee experience. In that sense, IAT makes more explicit an ecological perspective in tracing in a thematic manner the major disruptions in psychosocial support systems that the refugee has experienced through his or her trajectory of displacement. In each case, the emphasis may differ. For example, for some, a major concern may be the interpersonal difficulties encountered by the person's expression of anger and aggression, a pattern that may be traceable to a past history of recurrent exposure to acts of injustice (ADAPT Pillar III) occurring across several phases of the refugee trajectory. At the existential level, the person may be encountering difficulties in reconciling pre-established beliefs with the abuses they have experienced, making it difficult to retain a sense of faith in humanity and life in general, a problem located within ADAPT Pillar V. Existential doubts may be regarded as one of the ‘stuck points’ within CBT, whereas these concerns are seen as central to the refugee experience and a direct focus of IAT.

In the process of adapting IAT to each refugee group, background work is needed to ensure that the terminology used is culturally appropriate, ideally including indigenous metaphors to facilitate understanding of key concepts. Given that the emphasis of IAT is on facilitating a deep understanding of the sources of distress and the aim is to build adaptive capacity in a manner that is flexible and responsive to the changing environment, the expectation is that the approach may have an enduring effect on recovery in a setting where refugees are likely to encounter further and potentially novel challenges.

## Methods

### Development of the generic IAT treatment manual for refugees and survivors of mass conflict

The lead author (a clinical psychologist) and DS (a psychiatrist) at the School of Psychiatry, University of New South Wales, both with extensive experience in refugee mental health and psychotherapy, developed the template for IAT based on the ADAPT model. As far as possible the principles and theoretical perspectives offered by the ADAPT model with evidence-based techniques that support therapeutic change, the overall process being tailored to the culture and context. Iterative changes were made to refine the manual with the aim to achieve a high standard in relation to ensuring the theoretical coherence of the approach overall; practicalities of application including ease of translation and comprehensibility; harnessing the potential for cultural/contextual adaptability; and in framing the material and process in a way that would facilitate task-shifting to community workers with no previous training in mental health.

### Early modifications of the IAT manual amongst West Papuan Refugees

The initial field work was conducted amongst ten refugees trained as psychosocial workers, drawn from the displaced West Papuan community in Kiunga, Papua New Guinea (between January and March 2016). Kiunga is a remote border town where a substantial community of West Papuan refugees has resided for some years. We conducted four focus groups in which workers were asked to evaluate and assist in modifying the working manual to make it culturally and contextually relevant, based on six criteria: comprehensibility, usefulness, ease of application, ease of translation, contextual applicability and sustainability.

### Iterative adaptations and refinements of the IAT manual amongst refugees in Malaysia

Prior to adapting IAT for refugees from Myanmar, the in-country team (AKT, MAAM, SK) conducted an epidemiological study amongst the Rohingya group (January to June, 2017). The survey was conducted by a team of ten Rohingya researchers who were later selected and trained as IAT counsellors. The programme of research was based on a long-term engagement and partnership between the UNSW team and key stakeholders including UNHCR Malaysia, Perdana University, and the Rohingya Society, Malaysia.

Between October 2016 and September 2017, drawing on the experience and modifications of the IAT approach undertaken with the assistance of West Papuan refugees in Papua New Guinea, the treatment manual was adapted to the context and cultures of three refugee groups from Myanmar, the Chin, Kachin and Rohingya in Malaysia, the equivalent process being implemented subsequently amongst Rohingya living in refugee camps in Bangladesh. Table 1 describes the cross-cutting treatment strategies included in IAT. The approach was both systematic and based on extensive less formal interactions during the process of engagement with each refugee group as well as with NGOs and UNHCR officers in both Malaysia and Bangladesh. The process of cultural adaptation was guided by the framework of Bernal and Saez-Santiago (2006), supplemented by information generated by a targeted desk review of cultural idioms of distress and local terminologies conducted in relation to each refugee group in each setting (see a comprehensive review of the Rohingya refugees (Tay *et al*., 2019*b*)). The first step was the translation of all materials including the manual into the appropriate dialects with the assistance of bilingual mental health professionals. Accuracy and the cultural congruence of translations were enhanced by an iterative series of qualitative assessments. Feedback was sought via focus groups and reports by key informant interviews involving a range of community members and clinicians experienced in refugee mental health in each context. In the sections that follow, we focus specifically on our experience of adapting IAT to the needs of Rohingya refugees living in Malaysia.

**Table 1.** Cross-cutting treatment strategies included in Integrative Adapt Therapy

Cross-cutting treatment strategies	Simplified terms	Description
Psychoeducation	Introduction, building rapport and encouraging participation	Highlight the common experiences of all refugees Link programme to the ADAPT model Link the refugee experience to the five ADAPT domains Focus on building resilience and adaptive capacity to manage distress associated with the core refugee challenges, avoiding labelling mental disorders Provide information about the programme (duration, benefits, expectations)
Trauma narrative/*in-vivo* exposure	Story telling	Identity significant stressful life events and narrate these (traumatic) experiences in a coherent and chronological manner Link each set of events to each of the five ADAPT pillars where appropriate including safety/security, attachments, justice, role transition/identity and meaning Normalise feelings of fear and anxiety Understand the link between past trauma, present and future challenges, linking these events to the ADAPT model
Problem solving	Problem solving	Identity at least three problems according to each ADAPT domain and focus on the one(s) most preoccupied with Explore the underlying feelings of distress and reactions to each problem Explore coping methods, strategies and any existing barriers and/or perpetuating factors Brainstorm solutions and adopt a solution Commit to trying the solution in a step by step manner
Stress management	Relaxation	Apply strategies to manage stress: controlled breathing, progressive muscle relaxation incorporating locally salient metaphors and analogies
Emotion regulation	Managing distress	Apply emotion regulation strategies to deal with and build tolerance for distress associated with the disrupted ADAPT pillars Identify and label emotions/feelings using visually salient pictorial aids Normalise feelings of distress Accepting emotions/feelings and letting go without judgement Distancing self from unpleasant feelings
Cognitive reappraisal	Thinking in a different way	Understand thoughts, feelings and behaviour and how these are connected Identify and challenge unhelpful, negative thoughts/beliefs according to the experiences arising from the ADAPT pillars Understand and overcome gaps between expectations and reality with an emphasis on change in role transition and identity before and post-migration
Meaning making	A life worth living	Accepting the reality, recognizing and appreciating all small things in life Give hope Find meaning in life (what is worth living for, e.g. goals, dreams) Committing to goals and a life worth living
Behavioural activation (optional)	Getting active	Scheduling and engaging in pleasant activities
Strengthen social support (optional)	Social support	Identify trusted persons such as family members, relatives, close friends who the client can confide in Encourage and maintain regular contact with the trusted person Teach assertiveness and communication skills

Prior to the adaptation, the treatment manual was translated into Bangla, the closest written language to the Rohingya dialect which lacks a standard written format. Drawing on our prior experience of adapting IAT amongst West Papuan refugees, we aimed to test using a qualitative approach: (1) whether the basic tenets of the ADAPT model could be understood by the Rohingya and resonated with their experiences as refugees in Malaysia; and (2) whether the community showed a high degree of receptivity and affinity for the treatment in relation to its cultural congruence, capacity to bring meaning to their lived experiences of conflict and persecution, and relevance to the conditions of displacement confronted by refugees in that setting.

Two training workshops for the Rohingya team were conducted by AKT and two bilingual clinical psychologists (MAAM and SK), between July and September 2017.

### Pre-piloting modifications of IAT using focus groups of Rohingya refugees and trained counsellors

Following a systematic process of adaptation, we conducted five focus groups with Rohingya refugees, comprising five persons in each group (total *n*  =  25); in addition, one focus group was conducted with ten trained Rohingya IAT counsellors. Both focus groups were conducted in Bangla and Rohingya by two bilingual clinical psychologists (MAAM and SK).

In the focus group discussions, participants were presented with the basic outline and structure of IAT including the topics and techniques covered in each session. They were asked to reflect on and discuss the cultural and contextual appropriateness of the format and content and the adjunctive materials and tools. Facilitators engaged the participants in a dialectical interaction regarding the relevance and application of the ADAPT pillars and IAT to their own situation and to the experiences of those of their families and communities within the specific culture and context of displacement. During this process, a key emphasis was on assessing whether participants readily drew connections between the erosion of the pillars and their personal reactions to challenges and stresses at the emotional, behavioural, interpersonal and community levels.

Participants endorsed the relevance of the ADAPT pillars, being the spontaneous and extensive sharing of personal examples in which all the relevant pillars were invoked to explain feelings, behaviours and interpersonal that were personally troubling. There was a consensus in all groups that the five pillars, when considered together, were comprehensive in summarizing the challenges confronting refugees; in addition, the proposed techniques for addressing the problematic personal reactions arising from the erosion of the ADAPT pillars were readily understood and broadly endorsed as being of potential use.

### Piloting IAT in a community sample of Rohingya refugees in Malaysia

In the next step, the counsellors conducted a pilot study in which IAT was offered to 20 purposively sampled Rohingya refugees of mixed ages, gender and social position, under the supervision of the research team (AKT, MAAM, SK). Feedback sessions during this period led to further adaptations to the language, format and delivery of IAT. Specifically, the length of IAT was reduced from seven to six sessions, combining the first psychoeducation with the second safety/security sessions. A more flexible approach was introduced to allow an early decision to be made in therapy concerning which of the core pillars should be highlighted based on the participant using a visual grid of the ADAPT domains in which problems were prioritised. The grid provided the road map to guide the focus and sequencing of subsequent sessions. The visual representations and analogies used to illustrate the ADAPT model were all well received by participants with minimal suggested modifications. The language of delivery was simplified and de-medicalised further to emphasise the psychosocial aspects of IAT. In addition, emphasis was given to the partnership between counsellor and participant in which the two worked collaboratively to develop an understanding of the latter's life trajectory according to the ADAPT framework as the foundation for building the participant's capacity to adapt to the evolving context and challenges in their ongoing lives.

## Results

The qualitative data obtained from the focus groups assisted in the refinement of both the content and format of the manual developed and piloted with West Papuan refugees. For example, after several trials, it was agreed that the individual session length of 45 min was most appropriate. Throughout the process, attention was given to ensure the accessibility and comprehensibility of all aspects of IAT given that the approach was designed for use by lay counsellors, consistent with the principle of task-shifting. The language of the manual was simplified, and technical terms were replaced with colloquial expressions and analogies, for example, recognised terms such as ‘thinking differently’ was used for cognitive re-appraisal, and the metaphor of re-building or repairing the foundations of a house for the ADAPT pillars. A Problem Pie (based on the five ADAPT pillars) provided a further visual representation of the five inter-related domains that were undermined. This was supplemented by the Happy House built on a foundation of five pillars each reflecting an ADAPT domain. The motif conveyed the key message that recovery was an active process of rebuilding all pillars simultaneously. The aforementioned ADAPT grid allowed participants to consider several inter-related issues: (1) defining the contextual and individual challenges pertaining to each ADAPT pillar and the ecological and social consequences in each context associated with the erosion of that pillar; (2) the associated maladaptive reactions each person may be showing as a consequence of the erosion of one or more pillars; (3) internal and external barriers to making positive individual changes to address or mitigate maladaptive response patterns; (4) strategies and tools to achieve change and how each may best be used in focusing on priority issues; and (5) practical methods for practicing strategies for change, for example, using coping cards containing positive statements to prompt the person to take appropriate actions when these challenges (internal or external) arise. The treatment process of IAT is described in Table 2.

**Table 2.** IAT treatment process: applying cross-cutting treatment strategies to ADAPT-specific challenges

	Assessment and identity problem areas in relation to the ADAPT-specific domains		Explore, identity and address underlying stress and reactions associated with the antecedents/precipitants/perpetuating factors		Apply cross-cutting treatment strategies to build capacity for managing core challenges and distress across multiple adaptive systems
The ADAPT pillars	Precipitants	Perpetuating factors	Adaptive Stress Reactions (Tay *et al*., 2019*a*)	Comorbid disorders	Cross-cutting treatment strategies
Safety and security	Persecution, torture, abuse, intimate partner violence	Insecure residency, conditions of protracted displacement, threats of communal violence	Fear, anxiety, panic, nightmares, flashbacks, recurring thoughts, arousal	PTSD, depressive disorder, anxiety disorders	Psychoeducation, stress management, guided narrative, problem-solving, emotion regulation, cognitive reappraisal
Attachments	Traumatic losses, separation and displacement from homeland	Continuing separation from family, ongoing displacement	Sadness, isolation, suicidal ideation, preoccupations with deceased	Complicated bereavement, prolonged grief disorder, separation anxiety	Psychoeducation, stress management, guided narrative, problem solving, emotion regulation, cognitive reappraisal
Justice	Past and current human rights violations	No legal redress or mechanism to address past and current violations	Anger, resentment, sense of injustice, unfairness, moral injury	Intermittent explosive disorder, explosive anger	Psychoeducation, stress management, guided narrative, problem solving, emotion regulation, cognitive reappraisal
Role and identity disruptions	Post-migration stressors and deprivations	Transition and adjusting to new role and identity	Frustration, confusion, sadness, difficulties adjusting to life	Depression, adjustment disorder	Psychoeducation, stress management, guided narrative, problem solving, emotion regulation, cognitive reappraisal
Existential meaning	Mass killing, genocide, ethnic cleansing	Loneliness, isolation, limited access to spiritual, religious activities that confer meaning	Feelings of emptiness, sadness, isolation, suicidal ideation	Depression, suicidality, dysthymia	Psychoeducation, stress management, guided narrative, problem solving, emotion regulation, cognitive reappraisal, meaning-making

### Key areas in which the components of IAT were clarified and consolidated amongst Rohingya refugees

#### Establishing shared goals and a common understanding

The preliminary work indicated the need to establish clear goals and expectations at the outset of IAT. In so doing, the psychosocial effects of conflict and displacement at the individual, family and community levels were ordered by the participant and the bridges identified that demonstrated how the erosion of the ADAPT pillars impacted on all these levels, a principle emphasised throughout IAT. In that sense, participants recognised that the process of achieving durable individual adaptations will benefit the system as a whole, a principle that resonated strongly with the world view of collectivist societies such as the Rohingya.

#### Assessing comorbid mental disorders, local idioms of distress and adaptive stress

*Refugee Mental Health Assessment Package (R-MHAP)*: To assess comorbid mental disorders, we included key assessment modules of the R-MHAP, designed specifically for use with refugees (Tay *et al*., 2015*b*). The diagnostic modules include PTSD, major depressive disorder, generalised anxiety disorder (GAD) and prolonged complicated bereavement disorder.

Psychologists AKT and MAAM – the latter being fluent in the Rohingya language – iteratively revised the wording of criteria to make conventional mental health diagnoses to ensure congruence with the culture and, where possible, the integration of indigenous idioms of distress (Tay *et al*., 2015*a*). In all instances, the disorder categories and their constituent criteria were extensively discussed with community representatives (individual, in focus groups and informally) to ensure they were described and defined in language and concepts that made sense to participants. Psychological symptoms were framed in both cultural and adaptive or idiographic terms, in this instance, tailored to correspond to the Rohingya conceptualisation of the self (Tay *et al*., 2019*b*). In addition, all technical terms were translated into colloquial expressions or replaced with the local vernacular or culturally congruent terms (e.g. cognitive re-appraisal was called ‘thinking differently’, emotion regulation was called ‘managing distress’, existential meaning (Pillar 5) was called ‘living a meaningful life’). In reflecting both the etic and emic components of the therapy, the DSM-5 (American Psychiatric Association, 2013) based symptoms used to identify each of the four disorders were supplemented with relevant additional items and culturally specific expressions of distress.

#### IAT-specific treatment monitoring tools: Adaptive Stress Index and Adapt meter

As a monitoring tool specific to IAT, we developed the Adaptive Stress Index (ASI) which comprises five independent subscales – safety and security; bonds and networks; access to justice; roles and identities; and existential meaning – designed to measure therapeutic outcomes and progress in managing stress reactions associated with the ADAPT-related challenges (Tay *et al*., 2019*a*).

We followed a sequence of steps to adapt and validate the ASI across four culturally distinct groups including the West Papuans, Rohingya, Chin and Kachin (Tay *et al*., 2019*a*). Qualitative data provided broad support for the five ADAPT domains congruent with the culture and language of each group. Subsequently, a multi-step psychometric analysis based on item response theory produced a shortened and refined version of the ASI, retaining items with the greatest discrimination in defining the underlying ADAPT construct (Tay *et al*., 2019*a*). This method can be applied by teams working in similar settings to ensure the cultural and contextual congruence of the ASI. As a companion tool for counsellors to monitor therapeutic progress, to assess serially levels of distress associated with ongoing stressors posed by the ADAPT-related challenges.

#### Metaphors, illustrations, visual and pictorial representations

In terms of the use of concrete representations of distress, we used a picture of a glass filled with varying levels of water to depict higher or lower levels of intensity of emotions. The manual was embellished with metaphors including materials of specific cultural relevance, including stories, idioms and symbols to facilitate comprehension of the content. To ensure the concrete nature of the therapy, we used the story of a generic fictitious character (‘*Mohammed*’), a Muslim Rohingya man who fled persecution after experiencing extreme traumatic events in Rakhine state. He experienced great peril during the flight and conditions of severe deprivations after crossing the border. In this instance, the foundation of the ADAPT house was likened to bamboo pillars, a widely used construction material for Rohingya dwellings in Myanmar; the pictures indicated how, once damaged, the pillars can lead to the collapse of the house. Recovery is likened to rebuilding the house by repairing the damaged pillars in unison. In addition, culturally relevant pictures were incorporated into five sets of coping cards, each representing core challenges related to the undermining of a specific ADAPT pillar.

Low literacy levels amongst the Rohingya community required appropriate adaptations to the procedures used. For example, daily practice of adaptive techniques was prompted by pictorial representations on coping cards rather than by written materials. Further, coping cards with illustrations based on cultural expressions (co-designed with the IAT counsellors) based on the five ADAPT pillars are incorporated into the treatment to increase the comprehensibility of the content of the therapy.

Further information regarding the steps taken to increase the accessibility, uptake, acceptability and hence feasibility of IAT for the Rohingya community in Malaysia.

## Discussion

The aim of this report is to detail the rationale and iterative stages of early conceptualisation, development and adaptation of a novel psychotherapy, IAT, specifically designed to address the psychosocial and mental health challenges confronted by refugees. We outline a systematic staged approach in developing and adapting IAT first with West Papuan refugees and then amongst Rohingya refugees in Malaysia. The extensive adaptations and piloting described set the stage for an ongoing pragmatic clinical trial which is in progress in Malaysia.

While our approach to adapting IAT is based on a deductive framework drawing on the principles of the ADAPT model, the process of contextualizing and modifying the therapy to each group's culture, history and living context is substantially inductive, although remaining anchored in the informing theoretical framework. Specifically, although grounded in the ADAPT framework, in each setting extensive qualitative assessments are made in collaboration with the refugee community and service agencies, to ensure that the principles applied are localised according to the lived experience of the particular refugee group – as indicated by the procedures followed in modifying the approach amongst the Rohingya. The assumption guiding the approach is that there are universal principles and experiences that bring a level of commonality to the refugee experience worldwide but that these general sources of knowledge only make sense if contextualised within each culture and context.

## Conclusions

We have attempted to describe IAT in a manner that emphasises its novel elements as well as its overlap with established, evidence-based therapies in the field. This integrative approach – inherent in the naming of the therapy – aims to reflect an effort to harmonise several key elements relevant to the treatment field in this domain of practice. By grounding the method in the ADAPT model, we offer refugees a conceptual structure based on universal principles that appear to make sense when applied to the individual experience of being a refugee, providing a firm anchor point for developing mutual understanding between counsellor and participant. Importantly, the universal understanding offered by the ADAPT model is only useful if it is translated into an analysis of the practical challenges confronting each group based on the particularities of their histories, cultural understandings of mental health, and ongoing living conditions. Ultimately, the definition of problematic areas must be individualised to make sense of the needs of each participant.

In summary, IAT aims to integrate four essential goals: to provide a clear but flexible theoretical framework that makes sense of the refugee experience; to translate these principles into the real-life challenges and response patterns relevant to individuals within their culture and context; to use the understanding and insight gained as a motivator for individuals to make adaptive changes; and to provide them with the evidence-based techniques to develop adaptive strategies that are flexible enough to deal with ongoing and future challenges as they evolve.

Even though conducted at an individual level, in all phases of therapy, the scope of the therapy explicitly extends beyond the individual to a focus on the family and the wider community; in that sense, by the individual developing adaptive strengths, it is assumed that the benefits will flow on to help strengthen the ADAPT pillars at the systemic level, an intention that is made overt and which resonates with the members of collective societies such as the Rohingya. These core characteristics represent the novel aspects of IAT, offering an alternative approach to psychotherapeutic interventions for refugees. The adoption and wider use of IAT will await the outcome of ongoing trials, including amongst Rohingya refugees, examining the efficacy of the method.
